# Training Monitoring in Sports: It Is Time to Embrace Cognitive Demand

**DOI:** 10.3390/sports10040056

**Published:** 2022-04-08

**Authors:** Stéphane Perrey

**Affiliations:** EuroMov Digital Health in Motion, University of Montpellier, IMT Mines Ales, 34090 Montpellier, France; stephane.perrey@umontpellier.fr; Tel.: +33-4-3443-2623

**Keywords:** training, mental demand, effort, sport neuroscience, functional brain imaging, multidimensional, cortical activity, neuroergonomics, sports performance

## Abstract

Appropriate training burden monitoring is still a challenge for the support staff, athletes, and coaches. Extensive research has been done in recent years that proposes several external and internal indicators. Among all measurements, the importance of cognitive factors has been indicated but has never been really considered in the training monitoring process. While there is strong evidence supporting the use of cognitive demand indicators in cognitive neuroscience, their importance in training monitoring for multiple sports settings must be better emphasized. The aims of this scoping review are to (1) provide an overview of the cognitive demand concept beside the physical demand in training; (2) highlight the current methods for assessing cognitive demand in an applied setting to sports in part through a neuroergonomics approach; (3) show how cognitive demand metrics can be exploited and applied to our better understanding of fatigue, sport injury, overtraining and individual performance capabilities. This review highlights also the potential new ways of brain imaging approaches for monitoring in situ. While assessment of cognitive demand is still in its infancy in sport, it may represent a very fruitful approach if applied with rigorous protocols and deep knowledge of both the neurobehavioral and cognitive aspects. It is time now to consider the cognitive demand to avoid underestimating the total training burden and its management.

## 1. Introduction

Physical training causes biological, physiological, and biomechanical adaptations in athletes, which can improve sports performance [1,2]. This principle is usually reduced to a simple “dose-response” relationship [3] even if it is considered more complex than it seems due to non-linearities in the biological responses to training. It is well known that insufficient physical training leads to underperformance. On the contrary, excessive training could lead to the accumulation of fatigue and its concomitants (i.e., overreaching, overtraining). Consequently, this state may impair athlete performance and well-being while increasing risk of injury and illness [1]. Faced with this dilemma, coaches and sporting staff endeavor to determine the precise “dose–response” relationship between most effects provoked by training and athlete resources. When practicing sports, it’s well known that the brain (mind) and muscles (body) need to recover before individuals can perform at optimal levels. Within this context, physical health and mental health are two sides of the same coin. Increasing our understanding of the dynamic recovery—training balance is important not only because optimal performance can be achieved if athletes are able to cope with training stress with their own resources, but also because those adaptations influence athlete well-being and health. Thus, determination of athlete training burden is of great interest to sport practitioners and are widely used in the prescription and monitoring of physical conditioning programs [2].

Despite appearing to be important moderators of training, non-physical performance factors like motivation, anxiety, mental effort, and fatigue have received comparatively little attention compared to physical factors. In addition to a workout, other factors such as sleep, diet, and mental activity impact a body’s ability to train, recover, and perform. We can experience mental activity as effortful, and sometimes characterized by a lower willingness to engage in effort because of enduring exertion. Mental (or cognitive) fatigue is a psychobiological state caused by demanding cognitive activity relative to the mental effort and motivation required to perform a task [4,5]. Here, “mental effort” reflects the neurocognitive processes that control how much of an individual’s information-processing resources are actually allocated to the demands of one or several task(s) [6]. Mental effort is often considered as an invisible labor involved in training, and thus rarely considered in the management and planning for training. It may be because it poses many challenges for its accurate assessment. When dealing with training monitoring in sport environment, Halson [7] was likely one of the first to indicate assessment of cognitive function as one key feature to implement in a training monitoring system. Recently, Mellalieu et al. [8] emphasized relevant considerations for the conceptualization and measurement of what they defined “psychological load in sport” (i.e., the total environmental demands placed upon the individual inside and outside of sport). Commonly, load is referred as the net stimulus (that is ‘dose’) of a training session, combining fundamentally exercise intensity and volume indicators [9]. As load term is thought to be a problematic term [10], suitable terms were used thereafter in this review. Thus, volume and intensity of the training session might be lowered or increased with regards how individuals are responding and to result in their maximum performance. Monitoring the global and cumulative amounts of various burdens placed on an individual over time is essential to effective training management, adaptation, and injury mitigation in sports [11]. However, we can observe currently that training monitoring is mainly focused on the external physical stimulus applied to the athlete and its psychophysiological responses [9], offering a massive amount of data collected day-by-day by athletic staff. Although being complimentary and relevant, the use of data on mental effort has been very restrained in sports environments. While there is strong evidence supporting the use of cognitive demand markers in cognitive neuroscience and neuroergonomics fields (see next sections), their importance in sports science when considering training monitoring for multiple sports settings is quite neglected. Understanding and so monitoring the mental or cognitive demand in sports is fundamental and should complete the puzzle in training dose assessment and management.

The aims of this scoping review are to (1) provide an overview of the cognitive demand concept; (2) highlight the current methods for assessing cognitive demand in an applied setting to sports in part through a neuroergonomics approach; (3) show how cognitive demand metrics can be exploited and applied to our better understanding of fatigue, injury, and individual performance capabilities; and (4) give some perspectives on neuroimaging of the cognitive demand in sports. This scoping review opens up new thought on the relevance of multidimensional training monitoring. It is part of a line of thinking, considering the necessity to embrace cognitive demand within training monitoring approaches. Note that the current review was established on the search of several types of articles (online databases: Medline-PubMed and Google Scholar) that were centered on cognitive, mental, or psychological load/demand in the specific context of sports and training.

## 2. Definitions and Constructs of Training Burdens

### 2.1. Training Burdens

Let us consider first some definitions to clarify various constructs proposed in the literature about training monitoring. A variety of factors affect the body’s response to training dose. The latter is usually divided in two general constructs: the so-called internal and external training burdens. On the one hand, external training burden describes the physical work performed by the athlete during training workouts and competition. It refers to objective measures of training metrics such as training duration, total distance covered, number and magnitude of sprints or accelerations, mechanical power, speed, etc. [1,7,11]. External training burdens create physical, physiological and psychosocial demands, which are affected by frequency, intensity and duration of the exercise, among other factors; and all those demands may be sport-specific. On the other hand, the internal training burden refers to the individual internal responses (mechanical, physiological and psychosocial characteristics) to the external training burden. Importantly, internal responses vary over time, requiring ongoing monitoring. The range of internal burden measures includes several sub-components:

- perceptual (e.g., session rating of perceived exertion, sRPE [12,13]; and other psychological rating scales on the quality of the training sessions, recovery status, and wellbeing)
- physiological (e.g., blood lactate and heart rate—HR—at rest, exercise, and recovery epochs [1,9])
- biological (cortisol [14] for stress, creatine kinase for muscle damage marker [15])
- biomechanical (stresses and strains on the musculoskeletal system [16]).

Utilizing both training burdens is thought to provide a comprehensive view on whether an individual is in a state of “readiness” and able to tolerate high training dose, or in a state of “fatigue” and potentially at risk of injury or decreased performance. While measuring the external training burden helps to accurately “dose” an athlete is subjected to, the internal training burden helps to assess whether the response to the external training matches the intended response. Despite the association between external burden prescription and internal response, individuals may respond differently to the same external training dose due to multiple factors (e.g., age, sex, training, nutritional and psychosocial health status, body composition, history of prior injuries) [11,13]. This highlights the presence of various non-linear dose-response relationships when coping with acute or chronic training doses. As the internal training burden determines the functional training outcome, it should be used as primary measure when monitoring athletes. Hence, its monitoring is very important for understanding the demands placed on players, and for designing specific training sessions, injury prevention programs and recovery methods in sports environments [7,9,11,13].

Nowadays, many studies and sport team staff have adopted the internal-external training burden dichotomy when assessing training monitoring. However, this approach may be viewed as insufficient, given that other factors can affect the success of the athlete’s training session. A comprehensive monitoring should encompass mechanical (tissue stiffness), physiological (HR, blood lactate), psychological (motivation), social (coach-athlete relationships), behavioral (movement patterns) and cognitive (inhibition, attention, decision) factors. Consequently, a taxonomy based on objective and subjective indicators integrating complementary approaches in training monitoring with evidence-based training effects (biological, psychological, mechanical etc.) might be more valuable; all these indicators being observable and measurable. For instance, psychological, physiological, behavioral, and neurophysiological indicators belonging in part to the internal-external training burden constructs, can be used together for determining all the features of the cognitive demand. Even though it is accepted that external, internal, and cognitive demands might be separate constructs, Fuster et al. [17] indicated that they must be considered as a whole to optimize performance and prevent injuries. As such, the training burden is classically assigned as a multidimensional construct consisting of interrelated dimensions [9].

Interestingly, Coyne et al. [18] proposed a relevant and promising multivariate training model that considers physiological, biomechanical, and cognitive (mental) demands. The authors indicated that the mental demand appears to be an important moderator of the training burden for explaining performance and injury. Hence, the multivariate training model encompassing biomechanical, physiological, and cognitive demands was proposed by highlighting, in particular, sRPE as a useful subjective measure associated to changes in injury rates or performance. A call for action was even launched by Coyne et al. [18] on some issues for quantifying the ability to assess mental demand by using mainly current subjective measures. It was an important step, but likely insufficient, if we are looking closer to the cognitive demand concept in an applied setting.

### 2.2. Cognitive Demand

According to Williams and Ericsson [19], sport offers a valuable domain to explore the validity of models developed in other fields (e.g., cognitive neuroscience, psychology), because most sports require numerous higher-order cognitive functions and are involved under conditions of important stress where the limits of human behavior are being continually challenged. In their daily training environments, individuals are often exposed to sporting activities that are cognitively demanding. Cognitive cost induced by progressive mental activity when practicing sports solicits various cognitive functions. Sport situations requiring information processing are characterized by a high level of uncertainty constraining the player in team sport for instance to use a set of cognitive functions (attention, decision making, working memory) to give the most appropriate responses under stressful and demanding environments. Such cognitive functions are relevant in analyzing the ability to recognize an opponent’s action or in solving a problem. Results generally show that experts perform faster and more accurately on specific cognitive tasks [20].

Attention is the one of the important cognitive abilities in sport environment. Attention is described as conscious or unconscious focus of perception on a certain object, action, or activity [20]. Testing attention involves assessment of the ability to attend to stimuli, the ability to focus attention on selected stimuli, and ability to inhibit attention to inappropriate stimuli. Slowed reaction time is one of the most sensitive measures of impaired cognitive function. Reaction time refers to the time that passes between receiving a sudden and non-prefigured signal (auditory, visual, or tactile) to responding to this signal. An on-field measure of reaction time could be the time it takes to initiate movement after seeing an object (e.g., goalkeeper reacting to a penalty kick). In most sports, there are situations in which an athlete will need to use focused attention and when that same athlete needs to use divided attention. A soccer player will use focused attention when taking a penalty kick. The same player will use divided attention when he needs to perform more than one task at the same time. Being able to divide attention efficiently is helpful when the soccer player is dribbling down the field, sees an opponent approaching, and passes the ball.

Another executive function, response inhibition, is often associated with successful sporting performance in dynamic environments [21]. This is the case when individuals in interactive sports (e.g., handball) have to respond quickly to the actions of their teammates or opponents or frequently inhibit their already initiated responses (e.g., when reacting to feints). Also, athletes from open skill sports (i.e., changing and unpredictable environment as tennis) display superior response inhibition compared to non-athletes and athletes from closed skill sports (i.e., with constant and predictable environment as swimming) [22]. Specifically, superior inhibitory control is basically indicated by shorter stop-signal reaction times, meaning that individuals need less time to withhold their prepotent motor response [23]. Information recall is another cognitively demanding task. For some sports (e.g., soccer, tennis), this may be an opponent’s typical pattern of play, passing preference, or technical weakness. For other sports (e.g., cycling, skiing), this could be a previously analyzed competition track to help guide appropriate pacing.

In the situations described above, the inability to face to increased cognitive demand may be a key contributing factor to decreased performance and recurrent injury. The response to cognitively demanding tasks and subsequent mental fatigue is highly individual and is influenced by various factors such as the complexity of the task assigned, the cognitive functions solicited, the emotional state and expertise level of the individual. How to cope with excessive cognitive demand to maintain athlete performance is an emerging topic of growing interest [8,17,24,25,26]. However, this topic is still seldom addressed and implemented in training management, while coaches, support staff and athletes recognize it as a very important factor for performance. As proposed some decades ago, the effort expended by a person to accommodate task demands is a critical dimension of mental demand [27,28]. In terms of the effort encountered in sport environment, there are several related models, including the so-called “mental workload” [28,29,30], “cognitive load” theory [31], and mental effort [32]. Across these different terminologies, however, mental/cognitive demand may be described as the amount of mental effort required to execute a task within a limited time. Despite interest in the topic for the past 60 years in cognitive neurosciences, there is no clearly defined, universally accepted definition of the so-called “mental workload”. The latter as a mental construct, is multidimensional and results from the aggregation of many different demands. It refers to the amount of working memory processing a task in the restricted time [33]; working memory being an executive function that requires holding information and updating that information as needed to respond accurately to a subsequent task [34]. For clarity, cognitive demand term rather than “mental workload” is mainly used later.

Thus, cognitive demand is trying to quantify the amount of mental demand a task puts on the mental resources. Cognitive demand is also seen as the pressure put on human working memory while performing a task. Finally, a popular definition proposed by Hart [35] is “a hypothetical construct that represents the cost incurred by the human operator to achieve a particular level of performance”. The term cost refers to the idea that processing resources are limited and that successful performance on a task requires some of these resources. In other words, when the cognitive demand required by the task is lower than the available cognitive resources, the task will be performed accurately. On the contrary, when the cognitive demand exceeds the available resources, the task performance will be lowered. In fact, the concept of cognitive demand may be explained by many theories including working memory, cognitive, or attentional theories [36,37,38], meaning that, it might be helpful in the sporting environment to determine the neurocognitive status not only based on the test performance, but also on the mental demand and effort.

In sports, attention was brought the last few years to mental fatigue, a psychological state [39] caused by prolonged periods of demanding cognitive activity [40]. In this specific context, it refers to excessive cognitive demand and may in turn lead to increased risk of error, modulate decision making, etc. As stated by Van Cutsem et al. [41], mental fatigue has subjective, behavioral, and neurophysiological manifestations. Thus, all these components interacting together should be used and interpreted to assess properly cognitive demand in sports. Mental fatigue will not be scrutinized in the context of this scoping review; examining mental fatigue in the elite sporting environment is well detailed in the recent studies of Russel et al. [25,26] and in the review by Van Cutsem et al. [41]. Of note, in sports, it has been defined as “the cognitive demands placed upon athletes as a result of the environmental requirements and task constraints, and the interaction with an individual’s capacity to accommodate such loads” [26].

In contrast to the relative lack of research investigating cognitive demand in the sporting environment, changes in cognitive demand have been more extensively examined in other domains: military [42], transport [43] and medical [44]. A big obstacle for translating research from the lab to the field is the difficulty of quantifying cognitive demand. This is primarily due to the lack of means to accurately measure cognitive demand in an athlete’s training and/or competition environment. Recent developments have, however, demonstrated that such information might become more easily available in applied sport settings soon. In addition, other disciplines (Human Factors and Neuroergonomics primarily focused on optimizing human health and well-being; cognitive neuroscience) have considered more to fulfill this gap in sporting research. Their contributions in sports research have an important role in solving some issues related to training monitoring.

## 3. Towards Multi-Dimensional Training Monitoring

### 3.1. Current Indicators

Performance, behavioral, physiological, biochemical but also perceptual measures are all standard options used for athlete monitoring in sports. In the past few years, technological progress has led to the development of several monitoring devices, including wearable sensors, Global Navigation Satellite Systems (GNSS) trackers, power meters [45], inertial measurement units [1,11], and various apps. These innovative tools to monitor athletes instantly aim to gather information about training-specific underpinnings. Monitoring may serve the purpose of assessing whether an athlete is adapting and responding well to training program features. Monitoring may also aid in determining training and competition dose, particularly with intense competition timeline.

Basically, the methods used to measure exercise intensity can be either subjective or objective [1,11]. Common objective measuring tools include GNSS tracking devices that measure athlete movement and speed (i.e., external training indicator) and portable monitors to track HR and the rate of oxygen consumption (i.e., $\dot{V}O_{2}$, internal training indicator) during exercise. Noteworthy that external (absolute: power, acceleration, force) and internal (absolute: HR, $\dot{V}O_{2}$; relative: RPE, %HRmax or $\dot{V}O_{2}$max) indicators allow to define exercise intensity. Besides, exercise volume (distance, weight, repetitions) is quantified as the product of exercise intensity (speed, force), exercise duration (time) and frequency (exercise session or repetition). Subjective measuring tools, such as questionnaires or rating scales, are often used to gauge internal training burden, inviting individuals to record their rating of perceived exertion after training or competition (RPE) and their sense of wellness and wellbeing. The use of these psychometric self-report scales (available within minutes) allows to continuously monitor the athlete’s subjective experience of recovery and stress during the training process [18,46].

Due to the increased availability of wearable sensors, monitoring the external training burden appears still to be a priority in current monitoring practices by many sporting staff in high-performance programs, instead of focusing attention to other internal and cognitive training indicators than the self-report measures commonly used (e.g., sRPE, sleep scale). It means that training monitoring looks like it is more on a data-driven logic. This is especially true for team sports or cyclists that measure daily an important quantity of external (mechanical variables) and internal (HR) training metrics. While a large range of external and internal measures in training monitoring have been proposed this last decade, other factors request today to be considered and better captured. For instance, the use of psychomotor reaction time can be proposed as an indicator of cognitive demand. It has received very little attention in sports as yet.

To date all effort is put on a data-driven approach to training monitoring in sports but a data-informed approach may be more relevant. Gamble et al. [47] emphasized that practitioners and coaches should be data-informed rather than data-driven. In the current digital area, there is a need for further advancement towards more quantitative and qualitative training monitoring methods in multi-dimensional non-linear machine learning based models, integrating objective and subjective measures [48]. Athletes should be closely monitored to ensure the training process elicits the desired effects on athlete well-being and performance. Up to now, the available literature addressing responses to training sessions have examined multiple different indicators including biochemical, physiological, psychological, hormonal, but less related to the cognitive states of the individuals. Understanding the interaction of cognitive demand and movement patterns in the environment is a key component in performance outcomes. Cognitive factors interact with physiological and mechanical factors occurring during training and competition.

### 3.2. Cognitive Demand Indicators

As underlined before, cognitive demand is used to denote different factors and has many measurement methods associated with it. Being able to track cognitive activity in sport environment and using this data to make improvements to training management falls within the areas of neuroergonomics, defined as the study of the human brain function and behavior in relation to behavioral performance in natural environments and everyday settings. There is in fact a central research objective within neuroergonomics, that is assessing mental demand at work to develop aids and countermeasures [49]. Neuroergonomics is a combination of the Greek words neuro, meaning “relating to nerves or the nervous system,” and ergonomics, meaning “the study of work”—the study of brain and behavior at work. Sport is clearly of a suitable but not underlined and undefined applied domain for neuroergonomics. Neuroergonomics is significantly associated with mental states (e.g., mental demand, mental fatigue, etc.). The main contribution of neuroergonomics could be on the evaluation of mental demand related to cognitive training. Neuroergonomics has clearly benefitted from the committed inclusion of neuro-based methods and techniques, and it continues to develop in a variety of interesting ways [49,50]. There are several reasons why the so-called “mental workload at work” is critical in neuroergonomics. First, high cognitive demand of an individual can cause a state of distraction. Second, it can restrict the ability of an individual in a specific cognitive task since there is an individual limit to the number of cognitive resources. Consequently, the assessment of mental demand could play a key role in preventing sport injuries.

**Figure 1:**
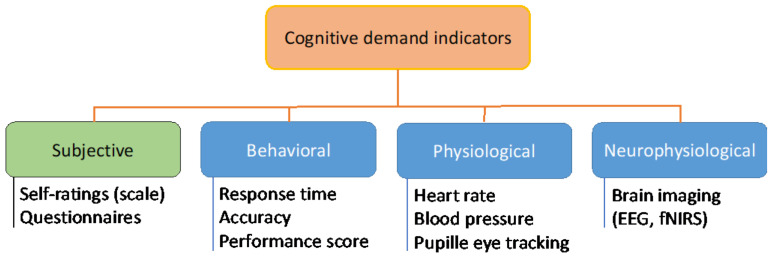
Summary diagram of all indicators of cognitive demand categorized in one subjective and three objective subdomains.

As for internal and external training indicators, there are a variety of subjective and objective metrics that it can be used for assessing cognitive demand (see Figure 1). Physiological methods to objectively measure cognitive demand have the advantage of being implemented continuously throughout, and independently of the task, unlike subjective measures that require individuals to self-report their experienced cognitive demand or cost at intervals or after the task.

#### 3.2.1. Self-Report Subjective Measures

First, as a psychological construct, cognitive demand can be assessed by various subjective measures by asking the individuals to rate their experienced cognitive demand on a rating scale immediately after finishing a task [32]. In Ergonomics, a unidimensional tool, the rating scale for mental effort consisting of a line with a length of 150 mm marked with nine anchor points (each label indicating a degree of effort) was considered sensitive enough to assess the subjective mental effort of tasks. Its applicability to sport activities remains to be determined. Of all the subjective measures, multidimensional or multi-scale ratings such as SWAT (subjective workload assessment technique, [51]) and NASA TLX (task load index [32]) appear the most common self-report indices of mental demand and are widely used within both neuroergonomics and human factors fields. Both allow aggregation procedure to produce an overall rating. The SWAT rates experiences on three dimensions (time pressure, mental effort and psychological stress) while the NASA TLX is built on six subscales (mental demand, physical demand, temporal demand, performance, effort and frustration). Although a weighting procedure (from 0 (very low) to 100 (very high)) exists for the scoring of each subscale, raw scores are often used because of ease of assessment. The NASA-TLX has proven its sensitivity in a variety of cognitively demanding tasks and domains (aviation, healthcare). The possibility exists to explore its utility in relation to assessing the cognitive demand associated with sporting performance [52,53].

Finally, a few studies exploited session ratings for cognitive and technical demands (cognitive RPE and RPE-T, respectively), based on the answer to the question “How much mental effort and decision-making has this task required?” Farrow et al. [54] showed that cognitive RPE was sensitive to motor tasks that involved increased decision-making in game-like situations. Open drills were more physically and cognitively demanding than the closed drills. Barrett et al. [55] observed positional differences for RPE-T following soccer match but indicated there were a number of confounding factors that might influence the individual’s level of perceived cognitive exertion. The review of Fuster et al. [17] suggested that cognitive RPE might be sensitive enough for open skill sports mimicking competition.

The pragmatic utility of self-report subjective measures is well established, but they have several limitations. First, especially in high-stakes settings, responses can be subject to biases. Second, self-reports are not well suited to continuous monitoring of cognitive demand. Even when subjective rating scales are applied repeatedly within a task, it is unclear if the subjective methods provide a continuous measure of fluctuations in cognitive demand during task performance [56]. Using objective psychophysiological indicators could overcome those limitations.

#### 3.2.2. Behavioral Measures

Behavioral measures such as performance from neuropsychological tests may be indirect measures of cognitive demand. Yet, similar to subjective measures, behavioral measures are not continuous. Response time and accuracy (i.e., percentage of correct or incorrect answers) during cognitively demanding tasks are often reported as objective indicators of mental demand experienced by the individual. Making a successful decision depends on the ability of the player to identify, select, and then execute the correct action in response to the movements of opponents or teammates, recognizing significant patterns in the game and determining the situational probabilities [57]. Decision-making time is determined as the time interval between the first identifiable contact of the stimulus-player and the first identifiable contact that initiates the participant’s response [58]. This cognitive ability to make fast and precise decisions is fundamental for success in team sports. Scanlan et al. [59] investigated the influence of cognitive factors on reactive agility performance in basketball. They found that cognitive measures (decision-making time together with reaction time) had the greatest influence on reactive agility performance in basketball players. Specifically, response time and decision-making time were correlated with reactive agility movement time. This highlights further the contribution of cognitive qualities to open-skill agility performance in basketball players. Gantois et al. [60] showed that mental fatigue impaired the decision-making process, provoking a decrease in performance. These findings agree with those of Smith et al. [61] and Trecroci et al. [62], showing that mental fatigue affected the decision-making precision and time in soccer players.

#### 3.2.3. Physiological Measures

Physiological measures have the advantage to assess cognitive demand or cost in-real time and can provide a continuous recording of data over time. Physiological measures stand on the assumption that as mental demand levels change, there will be a corresponding response in the autonomic nervous system which can be reflected in several physiological parameters. Electrodermal activity [63] and changes in facial temperature [64] are potential physiological measures that have evidence supporting their use as tools for distinguishing between mental demand levels. Cardiovascular measures such as HR, systolic blood pressure and diastolic blood pressure were found sensitive enough to cognitive effort [65]. However, HR measures suffer from interactions with respiration, physical work, and emotional strain. The autonomic nervous system is responsible for the regulation of the heart rate variability through parasympathetic and sympathetic modulation, the balance of which is disrupted after training [66]. Heart rate variability was shown to be very sensitive to task-related cognitive demands [67].

Task-evoked pupillary responses can also be used to provide an estimate of the cognitive demand required to perform a task. Changes in pupil dilation, measured by an eye tracker, might reflect effortful processes during cognitive demanding tasks [68,69] where pupil diameter generally increases with higher cognitive processing levels. Although ocular measures are sensitive to mental demands, they are also sensitive to other factors, such as emotional states, making it less diagnostic.

#### 3.2.4. Neurophysiological Measures

Brain imaging techniques as a measure of mental demand are growing rapidly in popularity [70,71,72,73]. Significant advance in technology this last decade has facilitated the use of electroencephalography (EEG) from the lab into more ecological settings. Electroencephalography remains the most commonly used technique for measuring mental demand [74]. Electroencephalography is a noninvasive technique where the electrodes are placed on the scalp to measure electrical activities for the human brain [75]. Electroencephalography measurements have high temporal resolution, but a relatively weak spatial resolution and can be susceptible to artefacts [75,76]. With EEG, movement related readiness potential and preparatory slow brain potentials seem to be complementarily sensitive to attention, demand, and decision making. In the frequency domain, gamma (>30 Hz) oscillations modulated by sensory input are linked to working memory, learning and attention [77], while theta oscillations have been associated with cognitive control and response inhibition [78]. The evidence provided in the review of Park et al. [79] indicates that developments in mobile EEG technology and progress in signal processing now make it possible to monitor brain activity during active sports performance, such as golf or cycling, without impeding the execution of movements. Limits do still apply in high-impact sports such as running. With high-density EEG recording, neuronal oscillations can be readily recorded in a non-invasive way in human, allowing the possibility to follow the dynamics of brain activity during complex movement for providing quantitative feedback (i.e., neural biomarkers of performance) to practitioners and coaches [80].

One complementary promising approach is the non-invasive measurement of brain activity using functional Near-Infrared Spectroscopy (fNIRS) [71,73,75,76]. With relatively good spatial and temporal resolution, fNIRS is also quite robust against motion artefacts [75,76,81]. The advances of multi-channels and portable fNIRS hardware have enabled studying functional brain (frontal lobe) adaptations during complex motor tasks such as juggling [82], slacklining [83], squatting [84], basketball [85], playing table tennis [86], climbing [87]), and cycling [88]. Hence, measuring brain activation seems achievable during the execution of sports-related movements and even during an outdoor activity in a real-life situation. Further limitations, as well as current contributions and possible prospects of fNIRS and EEG in sports setting, are discussed in a position article by Perrey and Besson [75]. The fNIRS uses near-infrared light to measure changes in blood oxygenation in the brain. Brain activity can be indirectly evaluated from this, based on the concept of neurovascular coupling in which active brain regions require increased blood flow to meet energy demands [81]. With increased mental activity, an increase in concentration of oxyhemoglobin and a slight decrease in levels of deoxyhemoglobin are expected [89]. As a non-invasive, portable and movement tolerant brain imaging method, fNIRS is perhaps the most effective technique for measuring mental demand in-the-wild with fewer protocols requirements in long time durations. Brain activation in the prefrontal cortex, an area associated with executive functions required for the cognitive processes can be used as a valid indicator of mental demand [71,90]. Neurocognitive research on individual effort showed that increased mental effort is frequently associated with increased brain activation, particularly in frontal regions [91].

Monitoring cognitive demand in sports with wearable non-invasive brain imaging methods coupling EEG and fNIRS [70] can be one of the core applications of neuroergonomics in the future [50]. There have been many original studies associated with new method detection and analysis of the mental fatigue in sports using EEG data [72,92,93]. In order to detect with a better accuracy some cognitive states based on EEG and fNIRS data in situ, recent studies moved toward diverse machine learning techniques including support vector machine [94,95], artificial neural network [96]. Novel deep learning techniques framework can be a good approach for detecting cognitive/mental demand [97,98] in sports environments.

## 4. Perspectives on Neuroimaging of the Cognitive Demand in Sports

Studying cognitive demand using brain imaging techniques started in uncontrolled environments, as encountered in sports. Several recent proof-of-concept studies conducted for the first-time online measurements of hemodynamic response alterations with fNIRS during sport activities.

Functional Near-Infrared Spectroscopy was used for the first time on the field to measure brain activity in soccer players taking penalty shots. Psychological factors, such as anxiety and pressure, are among the critical causes of the mistakes adversely influencing the quality of a penalty kick, commonly known as choking under pressure. Results from [99] showed that for players who tended to experience more anxiety and miss penalties, the prefrontal cortex and the left temporal cortex were more active. These brain regions are involved in long-term thinking, suggesting that such players were thinking about the consequences of missing the shot, which impaired their performance. This result supports evidence for the neural efficiency theory, where the “correct” regions of the brain need to be activated to effectively carry out motor tasks under mental pressure. Within a cognitive training, using fNIRS technology in a closed-loop brain-computer interface (neurofeedback techniques) could help players to perform better under pressure by showing how their brains are behaving. Task difficulty is known to be related to response time as well as effort investment, two aspects that may also account for differences in brain activation. Based on Brehm’s theory [100], effort expenditure directly depends on task difficulty and motivation. Highly skilled athletes normally perform with minimal effort compared to novices, as directed by the concept of economy from the brain activity, with an inverse relationship between optimal performance and consumption of resources [101]. In sports science, this mechanism is recognized as neural efficiency. Based on a study conducted by Ludyga et al. [102], cyclists with high level of maximum oxygen consumption showed less cortical activity due to inhibition of task-irrelevant cognitive processes. Based on expert-novice comparisons, recent evidence of the neural efficiency phenomenon most commonly observed for frontal brain areas with respect to sports performance has been documented [103].

Furthermore, the ability to measure neurocognitive behavior without being constrained led to increased research in analyzing the brain activity between two or more subjects, also called hyper-scanning [104]. Its application is currently focused on understanding the processes that factor into mental demand, decision-making within a team flow. It can benefit from wireless neuroimaging methods (fNIRS and EEG) as time progresses during the competition. We can imagine fNIRS-EEG-measured metrics of mental demand in team sport cooperation such as in Rowing.

Additionally, the mental demand quantification from brain imaging might be useful in strength training with biofeedback implemented in virtual reality [105], and the mental demand measured with EEG device could be used to modulate the training difficulty level [106]. In strength training, eccentric and concentric phases differ regarding their underlying cortical initiation and control during performance of typical whole-body movement, such as barbell squat [84]. Most sporting activities involve both eccentric and concentric movements (e.g., running, jumping) and a large proportion of our daily living activities require accurate control of eccentric movements (e.g., walking down stairs, sitting). Cognitive demand assessed by NASA-TLX (higher mental demand and frustration) and behavioral measures (slower choice reaction time and lower accuracy score) were found greater during an acute bout of eccentric than concentric cycling exercise [107]. Borot et al. [108] observed that eccentric cycling emphasizing a higher mental demand and recruited more activity in the frontoparietal network. Altogether, it suggests that eccentric movement presents a challenge in cognitive control system where the prefrontal cortex is playing a preponderant role independently of afferent input [109].

Training overload due to successive physical exercises over long periods without sufficient recovery may induce fatigue in the cognitive control brain system [110]. This chronic fatigue included reduced activity and downregulation in a portion of the brain (the lateral prefrontal cortex and middle frontal gyrus) important for making decisions. The findings show that, while endurance sport is generally good for health, overdoing it can have adverse effects on the brain. They draw attention to the fact that neural states matter: individuals do not make the same decisions when their brain is in a fatigued state. This is also true in team sports requiring attention for long periods before and during matches, adhering to tactical strategies, constantly adjusting to changes in the opposition and their teammates practice [111].

Capturing mental states with wearable brain imaging methods onto the training field has the potential to not only facilitate better understanding of the brain-behavior links, but also to produce new advances in sporting practice [111]. From a neuroscience perspective, measurement of mental state in situ has several potential advantages. First, the high degree of ecological validity would provide a more stringent test of the neural efficiency hypothesis. In addition, combined with other indicators (performance, psychological, biomechanical, physiological), longitudinal tracking of individuals over the course of training with EEG-fNIRS devices should facilitate a better identification of training-induced neuroplasticity and some differences related to the development of expertise across sports and between individuals [112]. Recently, several lines of evidence were revealed in a transversal study on brain structural and functional differences between endurance runners and healthy controls [113]. This neurodiagnostic approach [112] could be more implemented in training routines with wearable EEG-fNIRS devices to show up the important role of optimal brain processing on performance levels in competitive sports. While at this stage it is difficult to predict the full extent of such applications in sporting activities, it will promote sports neuroscience to deliver valuable insights into the relationship between psychological and physical aspects of sporting performance.

## 5. Conclusions

This review discusses the distinct aspects that are associated with measuring cognitive demand in sports. We hope that through this scoping review, sport scientists and practitioners will be able to critically consider the value and limitations of cognitive demand metrics and will keep pursuing new methods to measure this neglected burden in training monitoring. A detailed quantification is essential to better understand the cognitive brain processing that occur in sporting activities. The proposed neuroergonomics framework encompasses operationalizations of several indicators at the neurophysiological, psychophysiological, and behavioral levels of mental states linked to performance output that can be monitored continuously mainly in an objective fashion. Wearable non-invasive brain imaging EEG-fNIRS systems offer very promising opportunities for evaluating cognitive demand in sports settings, including the ability to track the brain activity of individual athletes, and even over extended periods of time.

## Data Availability

Not applicable.
